# On the construction of LIECE models for the serotonin receptor 5-HT$_{\text {2A}}$R

**DOI:** 10.1007/s10822-023-00507-3

**Published:** 2023-06-14

**Authors:** Aida Shahraki, Jana Selent, Peter Kolb

**Affiliations:** 1 Department of Pharmaceutical Chemistry, Philipps-University Marburg, Marbacher Weg 8, 35032 Marburg, Germany; 2 Research Programme on Biomedical Informatics, Hospital del Mar Research Institute and Pompeu Fabra University, 08003 Barcelona, Spain

**Keywords:** 5-HT$_{\text {2A}}$R, LIECE, Free energy of binding, G protein-coupled receptor

## Abstract

Computer-aided approaches to ligand design need to balance accuracy with speed. This is particularly true for one of the key parameters to be optimized during ligand development, the free energy of binding ($\Delta$G$_{\text {bind}}$). Here, we developed simple models based on the Linear Interaction Energy approximation to free energy calculation for a G protein-coupled receptor, the serotonin receptor 2A, and critically evaluated their accuracy. Several lessons can be taken from our calculations, providing information on the influence of the docking software used, the conformational state of the receptor, the cocrystallized ligand, and its comparability to the training/test ligands.

**Supplementary Information:**

The online version contains supplementary material available at 10.1007/s10822-023-00507-3.

## Introduction

The serotonin receptor 2A (5-HT$_{\text {2A}}$R) is among the main targets for therapeutics developed for the treatment of central nervous system disorders and it has been shown to be involved in the control of mood, emotion, cognition, and most psychiatric diseases. [1] The 5-HT$_{\text {2A}}$R belongs to the aminergic family of the class A G protein-coupled receptors (GPCRs). The serotonin (5-hydroxytryptamine; 5-HT) receptor family includes 14 receptor subtypes divided into seven major classes (5-HT$_{\text {1-7}}$). This classification has essentially remained unchanged since 1994. [2, 3] The 5-HT receptors are expressed throughout the human body, [4] and hence, associated with a multitude of physiological and pathophysiological processes. [1, 5] All the members of the serotonin receptor family become activated by the neurotransmitter serotonin. They are all GPCRs, except for the 5-HT$_{\text {3}}$ receptor, which is a ligand-gated ion channel. Modulation of the activity of 5-HT receptors is an attractive way to influence many diseases, e.g. depression, schizophrenia, bipolar disorders, and Alzheimer’s disease. [6] Therefore, and with an eye towards improving binding affinity and subtype selectivity of ligands of the 5-HT$_{\text {2A}}$R, many small molecules with different scaffolds have been designed. They can be categorized into three main classes: ergolines, tryptamines, and phenethylamines. [7] Members of the latter class usually show the highest selectivity for the 5-HT$_{\text {2}}$ subtype. [8, 9]

For such ligand and drug design campaigns, computational chemistry, molecular modeling, and docking calculations have become an indispensable part, accelerating the first step of drug development. However, the process of testing candidate small molecules, obtained from docking studies or medicinal chemistry approaches, in cell-based assays such as competition binding assays is a bottleneck, costly in both consumables and time. Thus, computational methods that are capable of estimating the free energy of binding of a molecule with high accuracy are of tremendous value in order to weed out non-binders as early on as possible. Nevertheless, an accurate calculation of the free energy of binding for docking poses with rigorous approaches that utilize alchemical methods, thus accounting for the intermediate states, is computationally very demanding and time-consuming. Hence, these methods are not suitable for application in a high-throughput manner.

Linear interaction energy (LIE) approaches as end-point methods [10], on the other hand, only take into account bound and unbound states of a protein-ligand complex. They therefore offer higher efficiency for predicting the free energy of binding. These semi-empirical methods are based on linear approximations and have proven to perform well for estimating the binding free energy in a comparably shorter amount of time, yet with acceptable accuracy. [11] In this method, molecular dynamics or Monte Carlo simulations are used to calculate the average of the free energy of binding of the bound- and unbound states. In an extension of LIE called “linear interaction energy with continuum electrostatics” (LIECE), the MD or Monte Carlo simulations are replaced with energy minimization and the explicit water with a continuum solvent model. This makes the method even faster and more efficient. [12] LIECE models have successfully been used for kinases, [13] but to the best of our knowledge not for GPCRs.

In the present work, we have utilized four sets of ligands of the 5-HT$_{\text {2A}}$R in order to derive LIECE models for a fast, yet accurate estimation of a ligand’s binding affinity. The data sets were chosen such that they cover known scaffolds of 5-HT$_{\text {2A}}$R ligands and span a range of molecular descriptors and affinity values. In addition, to reduce potential systematic errors, we paid attention to the experimental assays and chose only those ligands that had been tested in a radioligand binding assay with the same radioligand, [^3^H]-ketanserin in this particular case.

We strived to not just build models, but also determine possible reasons for their successful or unsuccessful performance. In particular, we wanted to investigate whether there is a clear correlation with how the underlying data was obtained.

While all the values for the different energy terms of each ligand were calculated with CHARMM, [14] we used two docking programs, DOCK3.7 and HYBRID, with relatively different algorithms for pose generation. DOCK3.7, as a physics-based docking method, utilizes anchor points inside the protein binding pocket, trying to fit the molecules to these points by considering the complementarity with a particular ligand atom. [15] HYBRID mainly relies on structural data of the protein and a bound ligand and biases each docked molecule’s pose to the binding mode of the cocrystallized ligand. [16]

Moreover, we investigated the effect of the receptor structure used in the docking calculations on the quality of the correlation between the experimental and predicted free energy values. To that end, we used two inactive conformations of the receptor based on PDB ID 6A93 [17], bound to risperidone and ketanserin, respectively, for all data sets. For one of the data sets, we also contrasted the docking calculations to the inactive-state models with similar calculations to two active-state models. One of the active-state models was based on PDB ID 6A93, bound to serotonin, and one based on PDB ID 6WHA [18]. We evaluated the resulting LIECE models in terms of the correlation R^2^ between the predicted and experimental $\Delta$G and the root-mean-square error (RMSE) between these values, and also analyzed a potential influence of receptor conformation, data set structure, and radioligand used in the assay.

We here present and discuss our results, which may serve as guidelines for the development of LIECE models for other GPCR targets.

## Results

**Table 1:** Parameters for selected LIECE models derived from each data set

	INT							no INT					
	$\alpha$	$\beta$	$\gamma$	$\delta$	R^2^	RMSE	$\langle \Delta \Delta G\rangle$ ^a^	$\alpha$	$\beta$	$\gamma$	R^2^	RMSE	$\langle \Delta \Delta G\rangle$ ^a^
DOCK-inactRisp													
NBPhe	0.171	$-$0.032	$-$0.054	$-$2.903	0.525	1.48	0.09	0.179	$-$0.029	$-$0.085	0.514	1.46	0.13
±std. dev.	0.028	0.010	0.017	0.712				0.029	0.010	0.020			
NBPhe-vdWonly	0.214	–	–	$-$3.596	0.485	2.71	1.90	0.314	–	–	0.485	2.95	2.40
±std. dev.	0.018			0.581				0.003					
All	0.057	$-$0.012	$-$0.022	$-$6.938	0.076	1.58	0.01	0.122	$-$0.011	$-$0.077	0.060	1.74	0.00
±std. dev.	0.003	0.001	0.002	0.164				0.003	0.001	0.009			
All-vdWonly	0.074	–	–	$-$7.354	0.064	1.59	0.00	0.274	–	–	0.064	1.96	0.00
±std. dev.	0.003			0.117				0.001					
HYBRID-inactRisp													
2Cdrugs	0.110	$-$0.004	$-$0.097	$-$0.616	0.673	0.82	0.06	0.112	$-$0.004	$-$0.104	0.673	0.82	0.02
±std. dev.	0.017	0.006	0.012	1.727				0.022	0.005	0.022			
2Cdrugs-vdWonly	0.087	–	–	$-$8.729	0.391	1.15	0.03	0.386	–	–	0.391	2.23	0.04
±std. dev.	0.006			0.194				0.003					
All	0.021	$-$0.023	$-$0.169	6.713	0.049	1.52	0.07	0.019	$-$0.024	$-$0.093	0.032	1.95	0.07
±std. dev.	0.002	0.002	0.015	1.484				0.002	0.002	0.002			
All-vdWonly	$-$0.009	–	–	$-$10.049	0.003	1.54	0.00	0.289	–	–	0.003	3.42	0.00
±std. dev.	0.001			0.019				0.000					
DOCK-inactKeta													
NBPhe	0.563	0.069	$-$0.080	13.727	0.812	0.99	0.02	0.225	0.003	$-$0.059	0.695	1.41	0.04
±std. dev.	0.036	0.008	0.014	1.561				0.030	0.010	0.020			
NBPhe-vdWonly	0.226	–	–	$-$3.202	0.451	1.69	0.06	0.317	–	–	0.451	1.80	0.00
±std. dev.	0.015			0.547				0.003					
2C drugs	0.148	0.014	0.043	$-$8.626	0.510	1.00	0.04	0.277	0.023	$-$0.026	0.461	1.06	0.02
±std. dev.	0.024	0.004	0.012	1.344				0.014	0.003	0.007			
2Cdrugs-vdWonly	0.131	–	–	$-$7.025	0.483	1.03	0.01	0.343	–	–	0.483	1.95	0.02
±std. dev.	0.007			0.246				0.002					
All	$-$0.020	$-$0.008	0.002	$-$11.004	0.011	1.61	0.00	0.166	0.014	$-$0.061	0.006	1.77	0.01
±std. dev.	0.004	0.001	0.003	0.210				0.004	0.001	0.002			
All-vdWonly	0.010	–	–	$-$9.646	0.002	1.63	0.00	0.267	–	–	0.002	2.32	0.00
±std. dev.	0.003			0.107				0.001					
HYBRID-inactKeta													
2C drugs	0.249	0.093	$-$0.017	6.063	0.631	0.92	0.06	0.229	0.072	0.026	0.613	0.95	0.06
±std. dev.	0.023	0.017	0.017	2.041				0.019	0.012	0.018			
2Cdrugs-vdWonly	0.159	–	–	$-$5.885	0.482	1.09	0.01	0.328	–	–	0.482	1.58	0.01
±std. dev.	0.010			0.038				0.002					
All	0.141	0.035	0.083	$-$10.363	0.251	1.52	0.01	0.124	0.033	$-$0.045	0.105	1.69	0.01
±std. dev.	0.005	0.001	0.006	0.044				0.005	0.002	0.003			
All-vdWonly	0.077	–	–	$-$7.799	0.099	1.66	0.01	0.294	–	–	0.099	2.30	0.00
±std. dev.	0.005			0.155				0.001					

$^{\hbox {a}}$ arithmetic mean of the difference between $\Delta$G$_{\text {pred}}$ for the full model and all the individual $\Delta$G$_{\text {pred}}$ values of the loo models

**Fig. 1:**
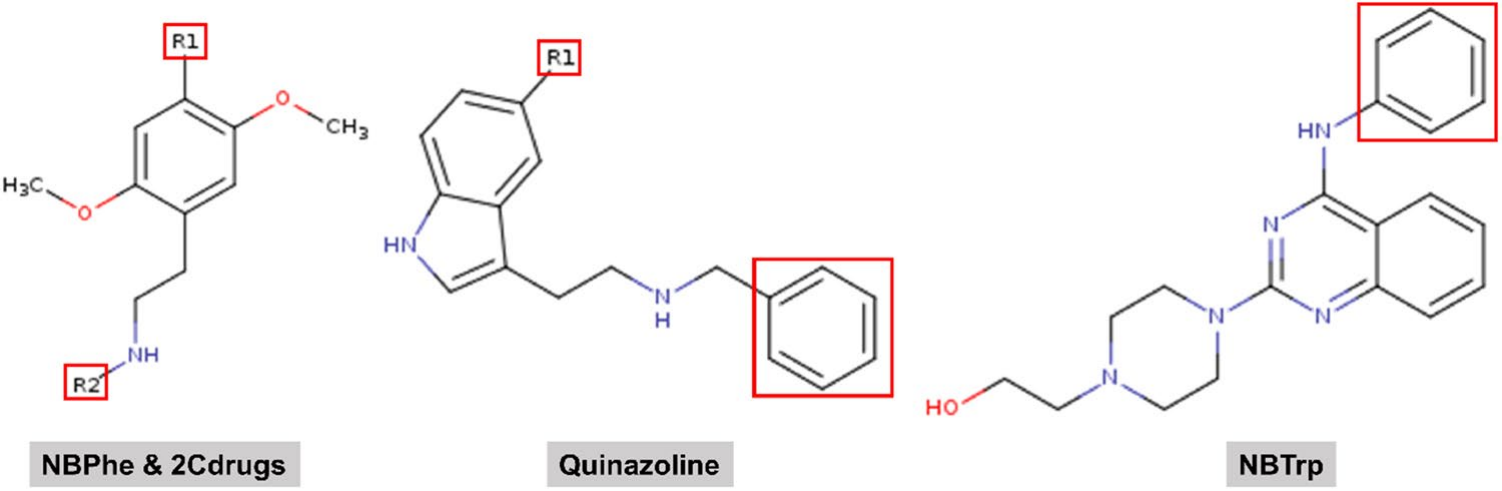
The 2D structures of the core scaffolds of the selected data sets. Red boxes depict the areas in which modifications have been introduced by the respective authors

To generate the LIECE models, we chose ligand sets that are based on known scaffolds of 5-HT$_{\text {2A}}$R ligands and for which affinity values are available. Figure 1 shows the 2D structures of the core scaffold of each data set.

We started the docking calculations using an inactive model of the 5-HT$_{\text {2A}}$R, termed ‘inactRisp’ in the following, prepared based on PDB ID 6A93 (cf. Methods). [17] We chose this structure because affinity values have been determined for risperidone at the wild-type receptor, allowing for an accurate calculation of $\Delta$G$_{\text {exp}}$ values for this ligand. Furthermore, because we were able to base our predicted binding modes on an experimental structure, we reasoned that the energy terms for the LIECE calculation can be determined with higher confidence than in cases where we do not have such a template.

Another point to consider was the experimental setup in which the affinity values were acquired and the radiolabeled ligand used in these, [^3^H]-ketanserin. We surmised that using a model with ketanserin as the cocrystallized ligand may be more relevant in our calculations because it is closer to the conformation that would be observed in real life. Hence, we repeated the docking calculations with an inactive-conformation model of the 5-HT$_{\text {2A}}$R bound to ketanserin, inactKeta (cf. Methods), using DOCK3.7 and HYBRID.

### Pose evaluation for the LIECE calculation

The pose evaluation step was the bottleneck of the process. This was true even though the 5-HT$_{\text {2A}}$R has a relatively well-defined pocket, in which the polar interaction with D155^3.32^ (the superscript indicates Ballesteros-Weinstein numbering [19]) is known to be necessary for receptor activation. Moreover, the role of other residues, such as S159^3.36^, S242^5.46^, F339^6.51^ and F340^6.52^, on regulating potency and efficacy of ligands has been studied extensively.  [17, 18, 20] Deciding on the correct alignment of molecules was not straightforward, as poses with an “upside down” orientation were generated as readily as canonical poses. The same was true for different orientations of flexible functional groups, each of which was present in various orientations.

**Fig. 2:**
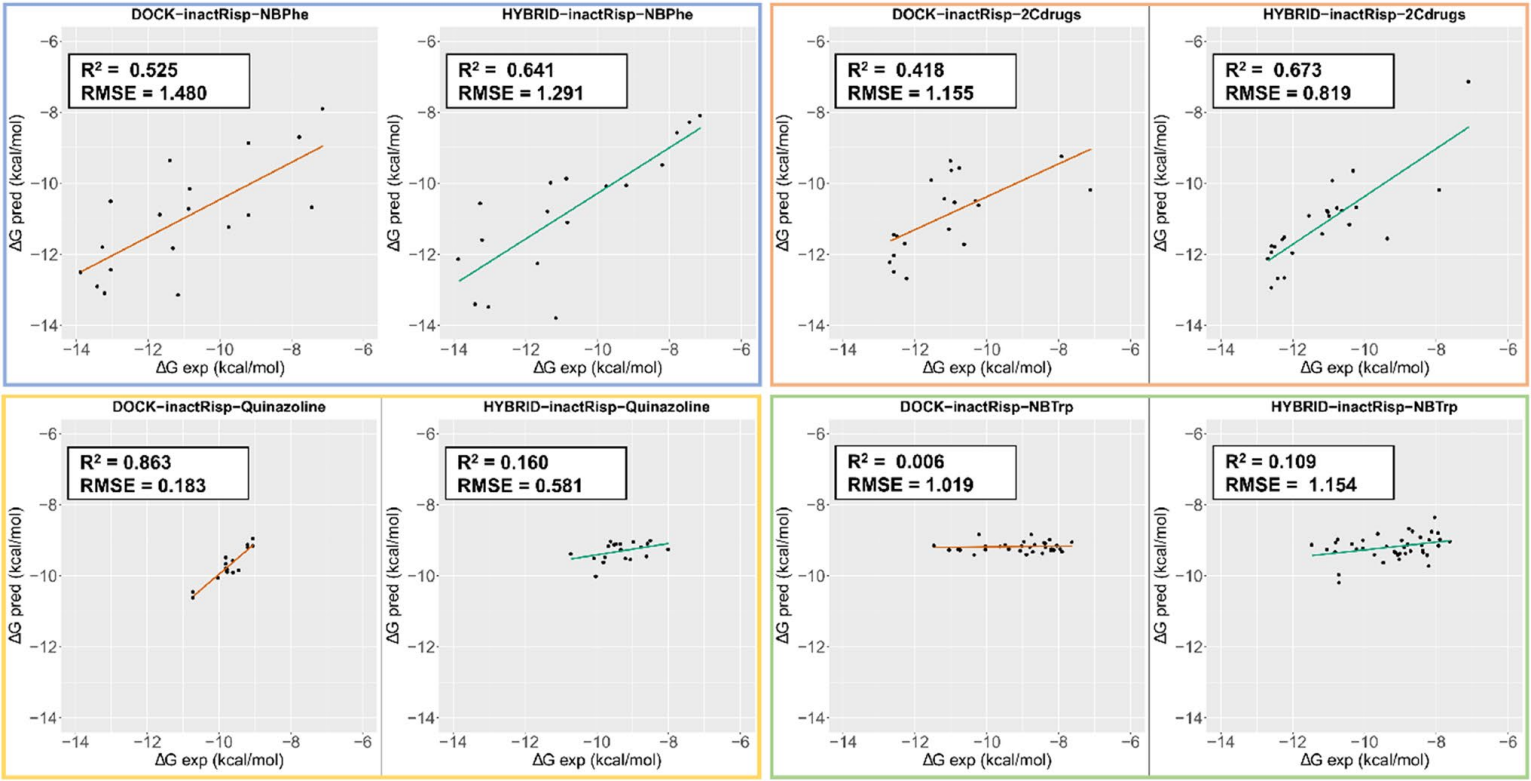
$\Delta$G$_{\text {pred}}$ vs. $\Delta$G$_{\text {exp}}$ plots for the LIECE models obtained for each data set with the inactRisp receptor model. Each colored frame groups the DOCK3.7 and HYBRID results for each data set. Color guide: NBPhe, blue box; 2Cdrugs, orange; quinazoline, yellow; NBTrp, green

For the purposes of this study, we tried to keep the poses within a data set as consistent as possible for comparability reasons, although this was not always possible. Poses that did not fulfill the constraints imposed on interactions by evolution and chemistry, i.e. no interaction with D^3.32^, stranded H-bond donors or acceptors, or solvent-exposed hydrophobic moieties, were not included in the LIECE calculations. For instance, no docking poses fulfilling these constraints were obtained for NBTrp in the inactKeta model when using HYBRID, and thus, this data set was not included in the LIECE calculations and analyses. Another example is the quinazoline data set, for which DOCK3.7 did not return any pose for a few of the molecules (apparent from the lower number of data points for the quinazoline data set in the DOCK3.7-related plots vs. the ones based on HYBRID; Fig. 2). However, if an interaction was possible in a slightly different orientation of the molecule in the pocket, in which for instance the angle or the distance between the donor and acceptor becomes favorable for an H-bond, then that pose was considered.

### LIECE models

The energy terms, including van der Waals (vdW), Coulombic, and desolvation, were calculated for the selected poses in CHARMM [14] and these terms were correlated to the experimentally obtained $$\Delta$$G ($$\Delta$$G$$_{\text {exp}}$$) values. Linear regression was performed to compute the correlation coefficients $$\alpha$$, $$\beta$$ and $$\gamma$$, respectively. The parameters were fitted with and without an intercept, $$\delta$$. Using these coefficients, predicted $$\Delta$$G ($$\Delta$$G$$_{\text {pred}}$$) values were calculated and LIECE models were generated for each data set. The linear regression coefficients, R^2^, and RMSE values for each LIECE model can be found in Tables 1, A1 and A2. We also monitored the stability of the LIECE models by employing leave-one-out (loo) cross-validation analysis. Based on this analysis, we calculated standard deviations (std. dev.) of the fitting parameters and the average of $$\Delta$$$$\Delta$$G$$_{\text {pred}}$$ (this is the difference between the $$\Delta$$G$$_{\text {pred}}$$ of each individual loo model and the $$\Delta$$G$$_{\text {pred}}$$ of the model considering all molecules of a set; $$\langle \Delta \Delta G\rangle$$). The parameters for models that we consider physically reasonable and of acceptable quality are presented in Tables 1. For comparison, values for models generated for all data sets are included in this table, too. The values for all other models are presented in Table A1 (with intercept) and A2 (without intercept).

Comparing the DOCK3.7 and HYBRID outcomes when using the inactRisp model, both softwares resulted in acceptable performances in the case of the 2,5-dimethoxy-substituted phenethylamines (2Cdrugs) and *N*-benzyl phenethylamine (NBPhe) sets. Superior RMSE and R^2^ values were observed for HYBRID-generated poses (R^2^ = 0.673 and RMSE = 0.819; Fig. 2). In case of the quinazoline set, the outcome of the two programs was very different. While the R^2^ value for poses from DOCK3.7 was 0.863, no correlation better than R^2^ = 0.16 could be obtained for HYBRID. RMSE values were 0.183 and 0.581 for DOCK3.7 and HYBRID, respectively, i.e. an overall low difference between $\Delta$G$_{\text {pred}}$ and $\Delta$G$_{\text {exp}}$. These two models are hard to compare, however, because DOCK3.7 was not able to generate poses for all of the molecules in this data set.

No correlation between $\Delta$G$_{\text {pred}}$ and $\Delta$G$_{\text {exp}}$ could be obtained for the *N*-benzyl tryptamine series (NBTrp) in any of the programs. The very low correlation obtained for this data set when using the two programs was surprising because both HYBRID and DOCK3.7 resulted in acceptable poses. The latter program achieved this to a greater extent, presumably due to the reduced emphasis on the structural input from the cocrystallized ligand. We speculated that this may have originated from the structural dissimilarity between the cocrystallized ligand in inactRisp, risperidone, and the NBTrp core scaffold, i.e. piperidine vs. tryptamine, or the state of the receptor. Therefore, we docked the same data set to two other 5-HT$_{\text {2A}}$R models; an active serotonin-bound model based on PDB ID 6A93 generated in our group using MD simulations (actSer), and a structure (termed ‘act25CN-NBOH’) prepared and modeled based on PDB ID 6WHA. The receptor is also in the active state in this structure and is bound to 4-(2-[(2-hydroxyphenyl)methyl]aminoethyl)-2,5-dimethoxybenzonitrile (25CN-NBOH). Docking calculations were performed with HYBRID to emphasize the structural information from the cocrystallized ligands. Despite reasonable poses in which the tryptamine moiety was placed at the bottom of the binding pocket, close to D^3.32^, S^3.36^ and S^5.46^, the correlations between the experimental and predicted free energy values were poor, R$_{\text {2}}$ = 0.049 and 0.021 for act-Ser and act25CN-NBOH, respectively (cf. Supplementary Figure A1).

Essentially, the same trend as for the inactRisp model was observed when we repeated the calculations using the inactKeta model (cf. Supplementary Figure A2), i.e. high R^2^ and low RMSE values for the 2Cdrugs and NBPhe sets and no correlation for NBTrp. For the quinazoline set, the difference to the R^2^ value which was observed with the inactRisp model became smaller, with DOCK3.7 still showing superior performance.

Lastly, we pooled all the energy values calculated for the four data sets by each docking program and generated a LIECE model based on the combined data to check if this would improve the correlation or not. When including all four data sets in the correlation studies, no satisfactory result could be obtained (Table 1, Supplementary Figure A3). We suspected that this may have been due to the negative contribution of the NBTrp set to the overall performance of the LIECE model (considering the poor correlation between the predicted and experimental for this data set). Therefore, we repeated the calculations with three data sets, NBPhe, 2Cdrugs and quinazoline (Supplementary Figure A4). Although the correlation between $\Delta$G$_{\text {pred}}$ and $\Delta$G$_{\text {exp}}$ improved in the case of DOCK-inactRisp and HYBRID-inactKeta, no R^2^ better than 0.25 was obtained.

As can be seen in Tables 1,  A1 and A2, in some cases linear regression coefficients do not follow the expected trend, i.e. they are negative for vdW and Coulombic energy terms and desolvation. This is particularly obvious when an intercept is included in regression analysis. Such negative coefficients are unexpected, as the energy terms themselves were negative for vdW and Coulombic energies and positive for desolvation. As a result, a negative coefficient would indicate an unfavorable contribution of the associated term. Looking closer into the data, we found out that $\alpha$ values are on average 10 to 11 times higher than $\beta$ and 2.5 to 3 times higher than $\gamma$ coefficients, suggesting that the main driver of ligand binding in the pocket of the receptor are the vdW interactions. When calculating the total electrostatic energy by adding up the Coulombic and desolvation terms, the resulting values are frequently very close to zero. This renders the corresponding coefficients, $\beta$ and $\gamma$, rather small and ill-defined. We hypothesize that in such situations, the fitting algorithm results in more or less arbitrary positive or negative signs of these coefficients. Hence, such models cannot be considered predictive. To test this, we repeated the fitting step by only considering the vdW energy, the corresponding parameters for which are included in Table 1 and Supplementary Table A1 and A2 (model names followed by "vdWonly"). If the electrostatic contribution is indeed negligible, this should yield models that are of comparable quality as the models based on all three energy terms. This was mostly observed, with models deteriorating in performance only marginally. Comparing the models in which an intercept was forced in linear regression with those without one showed the following differences: We found that the ‘INT’ models have improved correlation, whereas the ‘no INT’ models reflected the physical chemistry behind binding to a higher degree (i.e. had positive coefficients for vdW and Coulombic terms).

### Evaluating the quality of the LIECE models

We selected a set of 5-HT$_{\text {2A}}$R ligands with an NBPhe core structure as the test set. [9] The reason for choosing this scaffold was that the LIECE models based on NBPhe and 2Cdrugs showed the best performance in both inactRisp and inactKeta models (cf. Fig. 2, Table 1, Supplementary Figure A2, and Supplementary Tables A1 and A2). We repeated the same steps as previously explained during docking-based pose prediction and LIECE calculations for this data set and then used the coefficients obtained for each model, listed in Table 1, to compute the $\Delta$G$_{\text {pred}}$ values for the test set. The results are given in Supplementary Table A3. As expected, the models generated based on NBPhe and 2Cdrugs showed the best performance and the $\Delta$G$_{\text {exp}}$ values for NBPhe and 2Cdrugs overlap with the test set (Supplementary Figure A5). Specifically, the LIECE model generated for the 2Cdrugs data set using the inactRisp receptor model and HYBRID docking poses was noteworthy with RMSE values as low as 0.878. Due to the small span of the $\Delta$G$_{\text {exp}}$ values, however, R^2^ values were very low even for the best model (data not shown). In a ligand discovery setting, arguably the most important region of such a plot is the lower left region, which contains the correctly predicted high-affinity ligands. Many dots in that area, and a lack of such dots close to the x- and y-axis (which would constitute the false positives and false negatives, respectively) would make a model useful even though the numerical quality criteria might be considered suboptimal.

In addition, we wanted to test if any of our models could successfully predict the binding free energy for risperidone, for which affinity values for the wild-type 5-HT$_{\text {2A}}$R are available. Searching the literature, we found pK$_{\text {i}}$ values that range from 8.27 to 10, which equals $\Delta$G$_{\text {exp}}$ from $-11.27$ to $-13.63$ kcal/mol. [21–24] In the calculations we used the binding mode from PDB ID 6A93 and calculated the energy terms for this pose (cf. Supplementary Table A4). Mainly the quinazoline set-based models showed the best performance, predicting values in the same range as the $\Delta$G$_{\text {exp}}$. Comparing the chemical structure of risperidone with the molecules from the quinazoline set, we see that they share similarities, e.g. a pyrimidine ring in both, but there are also differences, such as a piperidine in risperidone vs. a piperazine in the quinazoline molecules. This may provide an explanation for the more precise $\Delta$G prediction for risperidone by quinazoline-based models — however, since the prediction is only for one molecule and not a set of data, no statistically valid conclusions can be drawn. Furthermore, for the quinazoline set, especially when using the inactRisp receptor model, the predictivity of the LIECE models was not consistent between different models and docking programs, limiting how useful they might be in practice.

## Methods

### Structure preparation

Two inactive models of the 5-HT$_{\text {2A}}$R were prepared based on the structure with PDB ID 6A93 [17] with the protein preparation menu of the Molecular Operating Environment (MOE) software. [25] In the first model, termed ‘inactRisp’, risperidone was kept as the cocrystallized ligand. To obtain a model with ketanserin as the cocrystallized ligand, ketanserin was docked to the 6A93 structure with DOCK3.7. [15] The top-ranked docking pose of ketanserin was then minimized using the MMFF94x variant of the Merck Molecular force field [26] in MOE. This structure will be referred to as ‘inactKeta’ hereafter.

Two structures of the 5-HT$_{\text {2A}}$R in an active conformation were used. One had previously been generated in our group based on PDB ID 6A93 following MD simulations of the receptor structure with a G protein to obtain an active state receptor bound to serotonin. The other one is based on PDB ID 6WHA [18], bound to 4-(2-[(2-hydroxyphenyl)methyl]aminoethyl)-2,5-dimethoxybenzonitrile (25CN-NBOH) which was prepared in MOE and then used in docking studies.

### Selection of the data sets

We searched published data sets to find those sets featuring ligands with scaffolds frequently occurring in 5-HT$_{\text {2A}}$R ligands and for which affinity values had been measured. To decrease the systematic error between the different data sets (that might lead to incomparability of the LIECE results), we ensured that all the ligands had been tested in the same experimental setup, i.e. a radio-ligand competition assay with [^3^H]-ketanserin. In the end, four data sets were chosen as the training sets for the LIECE models: *N*-2-methoxybenzyl derivatives of 2,5-dimethoxy-substituted phenethylamines (2Cdrugs); [27] *N*-benzyl-phenethylamines (NBPhe); [28]; *N*-benzyl-tryptamines (NBTrp); [29] and ligands with a quinazoline scaffold. [30] To test the quality of the LIECE models, a test set containing molecules with *N*-benzyl phenetylamine [9] scaffolds was chosen.

### 3D conformation of the ligands

The 2D structure of the molecules was sketched in the Marvin Sketch module [31] and saved in SMILES format. The.smi inputs were then used with two different 3D conformation generation approaches, OMEGA [32] and the db2-generation pipeline, for HYBRID and DOCK3.7, respectively. The 3D conformations of ligands were created at pH 7.4.

### Docking calculations

Docking calculations were conducted in DOCK3.7 [15] and HYBRID [16]. The templates, inactRisp and inactKeta, actSer and act25CN-NBOH were converted to the input format of the two docking programs, pdb and oeb.gz, for DOCK3.7 and HYBRID, respectively. All 3D conformers of all data sets were docked to the two inactive models using the two docking programs. Additionally, the docking calculations to the actSer and act25CN-NBOH were performed for the NBTrp data set. Three poses per ligand were generated and the best poses were inspected visually in Chimera [33] and PyMOL, [34] for DOCK3.7 and HYBRID, respectively. The selected poses were then saved in.mol2 format.

### CHARMM inputs

The CHARMM input files for the receptor models and the selected poses of the ligands were generated in the "PDB Reader & Manipulator" and "Ligand Reader & Modeler" menus of CHARMM-GUI [35] (https://www.charmm-gui.org/), respectively.

### LIECE calculations

The van der Waals and electrostatic interaction energies were calculated for the receptor alone, each ligand alone, and the receptor-ligand complexes in CHARMM [14], using the CHARMM46b2 force field. The estimate of the free energy of binding of each ligand was calculated by subtracting the energy values for receptor and ligand alone from the respective value for the receptor-ligand complex. In all calculations, structures were minimized in CHARMM with 500 steps of steepest descent mode followed by 1000 steps of the conjugate gradient algorithm, where the rms of the gradient was set to 0.001 kcal/mol. The van der Waals and finite-difference Poisson calculations were done on the minimized structures. The cutoff for the van der Waals energy was set to 14 Å. For the Poisson calculations, the exterior dielectric constant was set to 78.5, the one for the protein to 1.0 and the grid spacing for the focus procedure was set to go from 1.0 to 0.4. More details on the LIECE calculations can be found in refs. [12, 13].

### Binding free energy estimates

The reported mean K$_{\text {i}}$ values of the ligands were converted to the free energies of binding using equation 1, where *T* was set to 298 K.

1 $$\begin{aligned} \Delta G_{exp} = RT \cdot ln(K_{i}) \end{aligned}$$

The energy terms calculated from the CHARMM-based LIECE calculations were fit to a three-parameter model and a four-parameter version including an intercept (Eq. 2).

2 $$\begin{aligned} \Delta G_{pred} = \alpha \Delta E_{vdW} + \beta \Delta E_{coul} + \gamma \Delta G_{solv} (+ \delta ) \end{aligned}$$

where $\Delta E_{vdW}$ is the van der Waals interaction energy, and $\Delta E_{coul}$ and $\Delta G_{solv}$ correspond to electrostatic interactions and desolvation penalty, respectively. All these differences are calculated as $\Delta E_{complex} - \Delta E_{protein} - \Delta E_{ligand}$. Parameters $\alpha$, $\beta$ and $\gamma$ are the scaling factors. $\delta$ is the optional intercept. In our calculations, we examined the correlation between the predicted and experimental $\Delta G$ values, with and without considering the $\delta$ constant.

### Binding free energy estimates for the test set and risperidone

$\Delta$G$_{\text {exp}}$ was calculated with Eq. 1. The NBPhe test set was docked to the inactRisp and inactKeta receptor models using DOCK3.7 and HYBRID. LIECE calculations were run for the selected docking poses and the coefficients ($\alpha$, $\beta$, $\gamma$ and $\delta$) which were obtained for the various training sets (NBPhe, 2Cdrugs, NBTrp and quinazoline) were used to calculate $\Delta$G$_{\text {pred}}$ according to Eq. 2.

### Statistical analysis

The LinearRegression module of scikit-learn 1.2.1  [36] was used to fit the experimental and predicted $\Delta$G values using $\alpha$, $\beta$, $\gamma$ and the optional $\delta$ coefficients with a linear model. Cross-validations were performed using the LeaveOneOut module of scikit-learn 1.2.1. The R^2^ of the fit and the root mean square error (RMSE) between the predicted and experimental energy values were calculated in scikit-learn 1.2.1. The experimental vs. predicted $\Delta$G were plotted in ggplot2. [37]

## Discussion

Computer-aided drug discovery campaigns can benefit substantially from fast, yet accurate predictions of the free energy of binding. Despite the demand, such methods are still not broadly available and accessible in the field. Currently, end-point methods such as the LIE approaches are positioned between the computationally demanding but accurate rigorous alchemical methods and fast but less accurate docking scoring methods. Hence, they offer a balance between accuracy and speed.

Here, we have used a modified version of the original LIE method, LIECE, which substitutes MD simulations with energy minimization steps and explicit solvent with a rigorous treatment of continuum electrostatics. The success of the LIECE calculations for kinases [12, 13, 38] triggered the current study in which we sought to investigate the performance of this method for estimating the free energy of binding for GPCRs.

We ran LIECE calculations for four data sets comprised of ligands with known core scaffolds of 5-HT$_{\text {2A}}$R ligands. The affinity values for these molecules had been measured in radioligand binding assays, competing with [^3^H]-Ketanserin, and were used to calculate $\Delta$G$_{\text {exp}}$ values. Following the LIECE calculation and obtaining the vdW and electrostatic energy terms, the coefficients for a three-parameter equation (Eq. 2), with and without considering an intercept ($\delta$), were acquired through linear regression methods.

To account for the effect of different cocrystallized ligands – and thus slightly different binding sites – on the performance of the LIECE models, we used two inactive-conformation models of the receptor, bound to risperidone and ketanserin, respectively. The reason for preparing a receptor structure with ketanserin was to mimic the experimental setup in which the molecules needed to compete with ketanserin and bind to the receptor in this conformation (ketanserin-bound conformation). Furthermore, to inspect the effect of the different docking algorithms on the LIECE models, we used DOCK3.7 and HYBRID as docking engines. These two programs have slightly different approaches to including the structural information of a cocrystallized ligand when generating poses. This can lead to substantial differences between the docking poses and hence the accuracy of the LIECE models.

Our results did not reveal any significant differences between the LIECE models generated with the two receptor structural models. The overall performance of the LIECE models was acceptable for two out of the four data sets, namely NBPhe and 2Cdrugs. As for the effect of the docking program on the quality of the LIECE models, the results were comparable for both softwares, as they both resulted in similar trends for the different data sets. However, there were two cases with dramatic differences between the results based on HYBRID or DOCK3.7 docking poses: (i) LIECE models for quinazoline using inactRisp showed a significant difference between the R^2^ values for the two programs (0.8 for DOCK3.7 vs. 0.1 for HYBRID). This, indeed, happened as a direct consequence of the docking program: Lower numbers of successfully docked ligands, yet apparently more congruent docking poses, for DOCK3.7, in contrast to a higher number of poses, yet with lower consistency, for HYBRID. (ii) No LIECE model could be generated for NBTrp using the inactKeta model with HYBRID, since the program failed to generate any poses with the expected binding profile (interaction with D155^3.32^ and other residues of the orthosteric binding pocket of the 5-HT$_{\text {2A}}$R). These observations emphasize the importance of considering the similarity between the cocrystallized ligand and the ligands in the data set when using programs such as HYBRID.

At the other end of LIECE performance, the models generated for the data set with an NBTrp scaffold did not perform well in any of the tested conditions. In spite of testing additional different conditions for this data set, which included performing the calculations on the active state of the receptor with two different bound ligands, no correlation better than R^2^ = 0.14 could be obtained. Despite our best efforts, we were not able to find an explanation for this failure.

When testing our models using a test set with an NBPhe scaffold, we observed that the models generated based on ligands containing this scaffold provided the best correlation. This result emphasizes the importance of the similarity between the training and the test sets.

To elaborate on the potential errors in our calculations that might have resulted in low correlations between the $\Delta$G$_{\text {exp}}$ and $\Delta$G$_{\text {pred}}$ values, we should mention the questionable accuracy of the docking poses in some cases where no clear-cut decision could be made when choosing the correct binding mode. We believe that despite all considerations during docking pose evaluation, there might still be incompatible poses, which is reflected in the correlation plots. It should be mentioned that for any docking program, an average RMSD of 2-3 Å is expected between the poses and the experimental binding modes. [39] Such deviations can of course lead to less relevant binding energy values.

One other major source of error that leads to less accurate prediction of $\Delta$G values is the absence of water molecules. In fact, although often ignored in docking campaigns, water molecules can have a substantial effect on binding events and facilitate the binding of a molecule to the receptor through, for instance, water bridges. In our study, we did not consider any water molecule in the binding pocket as no such molecule was available in the used structures, 6A93 and 6WHA.

The other underlying reason for the occasionally low correlation between the predicted and experimental $\Delta$G values may be the flexibility of GPCRs. Compared to other protein families such as kinases, for which LIECE models resulted in high correlations and low RMSE values, GPCRs are more flexible by nature. In fact, GPCRs are highly allosteric proteins and their conformation can change quite dramatically in the presence of different extra- and intracellular binding partners, including small molecules, solvents, transducers such as G proteins, phospholipids, and cholesterol. Therefore, each set of related molecules might indeed bind to subtly different conformations of a receptor, thus rendering our assumption that we are able to treat the $\Delta E_{protein}$ values as constant moot.

Although with the tested data sets, we were not able to obtain a *universal* LIECE model for 5-HT$_{\text {2A}}$R ligands, we were able to clarify some of the conditions in which such models work and also address a few of the challenges. As a direct conclusion of our study, it seems more reasonable to generate LIECE models for each data set, choosing a suitable receptor structure based on the training set, and not striving for universal LIECE models.

## Conclusion

We have investigated the applicability of the LIECE method for the estimation of the free energy of binding for 5-HT$_{\text {2A}}$R, as one of the main targets for drug development for central nervous system disorders. We chose four data sets with representative core scaffolds of 5-HT$_{\text {2A}}$R ligands and, following a careful pose evaluation step, generated LIECE models for each data set. The evaluation of the correlation between the $\Delta$G$_{\text {exp}}$ and $\Delta$G$_{\text {pred}}$ values and the resulting RMSE revealed that our approach was successful in generating predictive LIECE models for two out of four studied data sets, namely NBPhe and 2Cdrugs. For these sets a consistent trend using different receptor models and docking programs was observed. The predictivity of the LIECE models for these two data sets was further assessed with a test set based on the same core scaffold.

For the quinazoline derivatives, DOCK3.7 outperformed HYBRID, and inactKeta appeared as the receptor model leading to more predictive models for this data set. We reason that this might be due to the fact that ketanserin also contains a 1H-quinazoline-2,4-dione group in its structure, further emphasizing the point that the training data should be as close as possible to the molecules for which predictions are made. However, we believe that the LIECE models for this data set need to be improved by including more data points, i.e. bigger training sets. In the case of the NBTrp scaffold, we failed to achieve a reasonable correlation between the $\Delta$G$_{\text {exp}}$ and $\Delta$G$_{\text {pred}}$ values, despite testing different conditions. This process will therefore need to be repeated with more NBTrp training sets as they become available in the future.

In conclusion, we demonstrated that upon careful selection of a suitable receptor model for the docking studies and the docking program itself, predictive LIECE models are achievable. However, to create such models, multiple conditions – including the conformation of the receptor, the cocrystallized ligand, and a suitable docking program – need to be tested. Along these lines, our study will be expanded when more structural information of the 5-HT$_{\text {2A}}$R becomes available. Moreover, one might consider different conformational states of the receptor in the future, to see whether more accurate estimates can be obtained. Of course, in prospective ligand discovery programs, it is hard to predict precisely which conformation is optimal.

Nevertheless, we believe that due to the flexibility of GPCRs, LIECE models with high correlation and low RMSE between $\Delta$G$_{\text {exp}}$ and $\Delta$G$_{\text {pred}}$ are still very challenging to achieve. Longer energy minimization steps, flexible docking, the explicit consideration of (bridging) water molecules or short MD simulations may indeed be necessary to account for the slightly different conformational arrangements in the binding pocket of the receptor.

## Supplementary Information

Below is the link to the electronic supplementary material.

Supplementary file 1 (csv 11 KB)

Supplementary file 2 (pdf 2337 KB)

## Data Availability

All calculated energy values are available in .csv format as Supplementary Information to this article. Additional $\Delta G_{exp}$ vs. $\Delta G_{pred}$ plots are found in the Supplementary Information. Docked complexes of all ligands can be found on ZENODO at 10.5281/zenodo.8028480.
