# Supplementary material for: On the construction of LIECE models for the serotonin receptor 5-HT2AR
Source: J Comput Aided Mol Des. 2023 Jun 14;37(7):313–23. doi: 10.1007/s10822-023-00507-3 (PMC10276788; doi:10.1007/s10822-023-00507-3)
Supplement: Supplementary file 2 — (pdf 2337 KB) [file 10822_2023_507_MOESM2_ESM.pdf]

Appendix A Supplementary Information

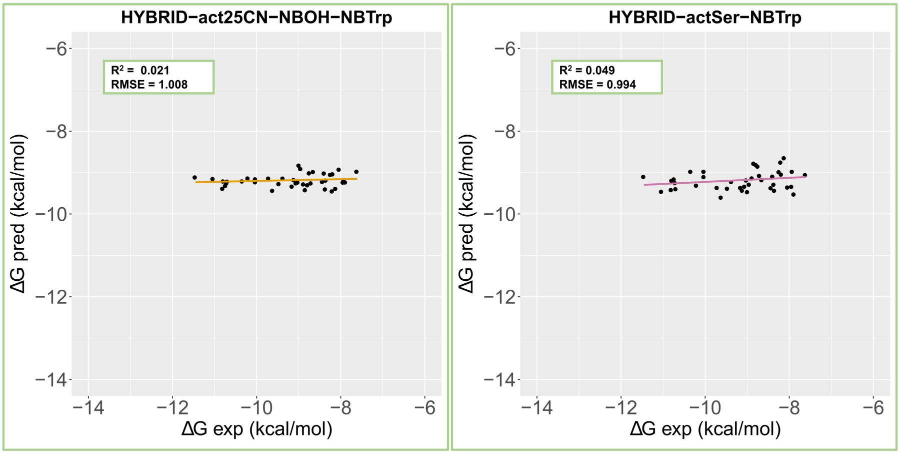

Fig. A1 ΔG_{pred} vs. ΔG_{exp} plots for the LIECE models obtained for NBTrp using act25CN-NBOH model (left) and actSer (right).

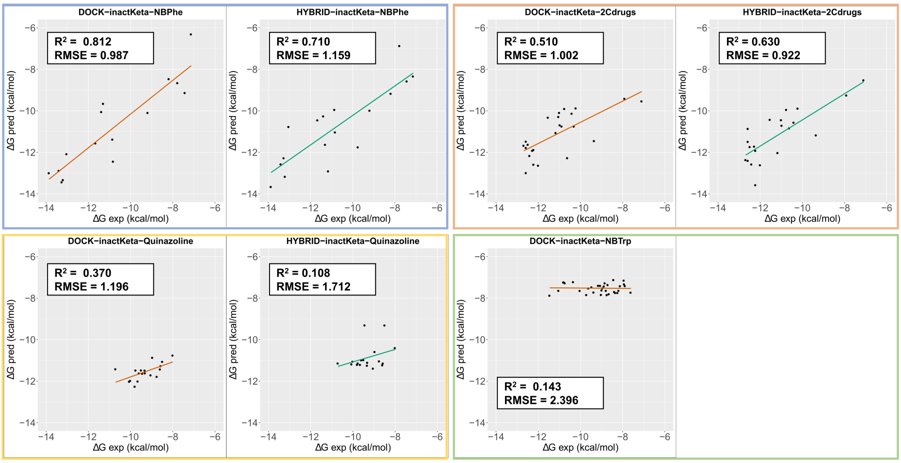

Fig. A2 ΔG_{pred} vs. ΔG_{exp} plots for the LIECE models obtained for data sets using the inactKeta receptor model. Color guide: NBPhe, blue box; 2Cdrugs, orange; Quinazoline, yellow; NBTrp, green.

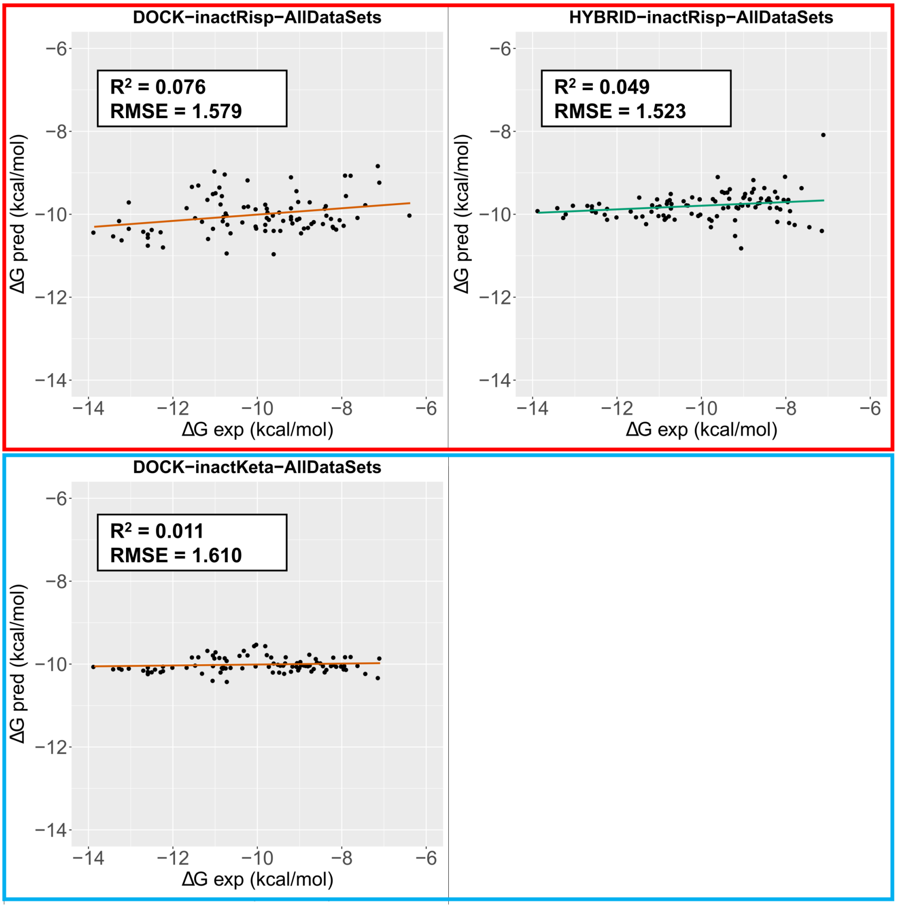

Fig. A3 ΔG_{pred} vs. ΔG_{exp} plots for the LIECE models obtained from pooling all the data sets together. The plot could not be generated for HYBRID-inactKeta as in this condition no plausible pose for the NBTrp data set could be obtained. The red and blue boxes depict plots for DOCK3.7 vs. HYBRID with inactRisp and inactKeta, respectively.

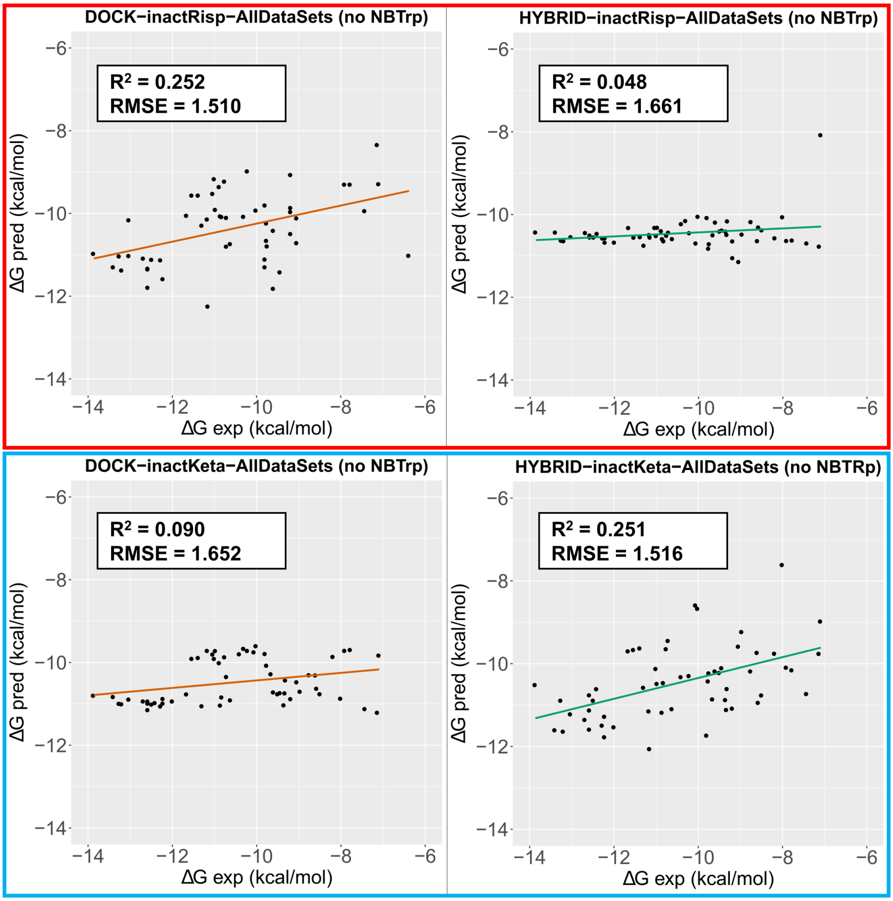

Fig. A4 ΔG_{pred} vs. ΔG_{exp} plots for the LIECE models obtained from pooling NBPhe, 2Cdrugs and quinazoline. The NBTrp data set was not included in the correlation investigations. The red and blue boxes depict plots for DOCK3.7 vs. HYBRID with inactRisp and inactKeta, respectively.

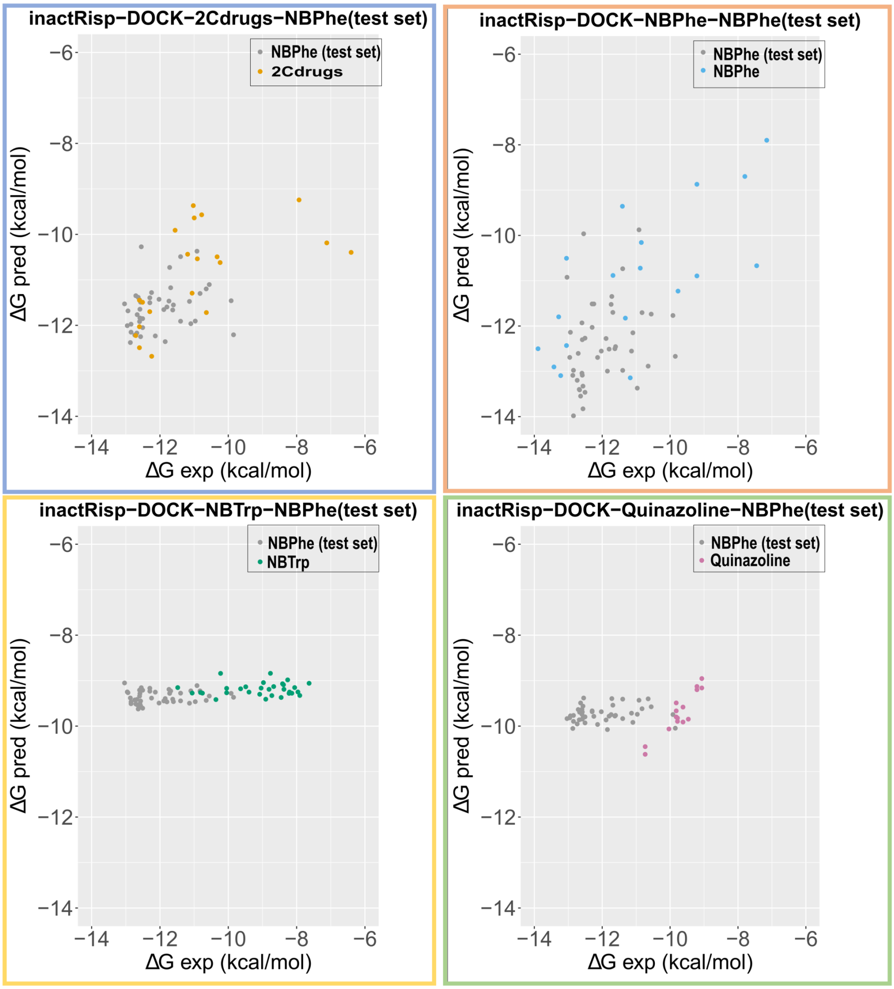

Fig. A5 The overlayed ΔG_{pred} vs. ΔG_{exp} plots for the four different data sets and the test set, NBPhe. Color guide: NBPhe, blue box; 2Cdrugs, orange; Quinazoline, yellow; NBTrp, green. The shown data belongs to the condition in which inactRisp receptor model is used for docking calculation with DOCK3.7.

Table A1 Parameters for other LIECE models derived from each data set, when forcing an intercept.

	**INT**						
	α	β	γ	δ	R^2	RMSE	$\langle\Delta\Delta G\rangle^a$
**DOCK-inactRisp**							
**2Cdrugs**	0.029±0.042	−0.022±0.017	−0.109±0.025	−2.571±0.419	1.001	1.16	0.10
**2Cdrugs-vdWonly**	0.116±0.011	/	/	−6.891±0.375	0.218	1.10	0.01
**NBTrp**	0.013±0.009	−0.006±0.005	0.013±0.007	−10.319±0.561	0.006	1.02	0.03
**NBTrp-vdWonly**	0.024±0.008	/	/	−8.288±0.288	0.000	1.03	0.01
**Quinazoline**	−0.017±0.004	−0.010±0.001	−0.044±0.002	−7.347±0.132	0.863	0.18	0.01
**Quinazoline-vdWonly**	−0.013±0.006	/	/	−10.171±0.256	0.013	0.69	0.01
**HYBRID-inactRisp**							
**NBPhe**	−0.002±0.019	−0.041±0.018	−0.412±0.026	18.233±1.902	0.641	1.29	0.09
**NBPhe-vdWonly**	0.092±0.023	/	/	−8.065±0.766	0.075	2.07	0.07
**NBTrp**	−0.067±0.006	−0.029±0.002	−0.008±0.007	−12.833±0.557	0.109	1.15	0.00
**NBTrp-vdWonly**	−0.056±0.007	/	/	−10.967±0.210	0.064	0.99	0.01
**Quinazoline**	−0.010±0.001	−0.006±0.002	−0.004±0.007	−9.544±0.474	0.160	0.58	0.02
**Quinazoline-vdWonly**	−0.011±0.001	/	/	−9.649±0.031	0.097	0.60	0.01
**DOCK-inactKeta**							
**NBTrp**	−0.060±0.018	−0.026±0.002	−0.032±0.005	−9.698±1.610	0.143	2.40	1.89
**NBTrp-vdWonly**	0.026±0.014	/	/	−8.232±0.527	0.002	1.06	0.01
**Quinazoline**	−0.056±0.019	−0.001±0.002	−0.048±0.005	−8.406±0.730	0.370	2.30	2.24
**Quinazoline-vdWonly**	−0.058±0.013	/	/	−11.653±0.530	0.097	0.62	0.01
**HYBRID-inactKeta**							
**NBPhe**	0.165±0.024	−0.081±0.013	−0.255±0.028	9.480±1.930	0.711	1.16	0.06
**NBPhe-vdWonly**	0.263±0.018	/	/	−2.366±0.583	0.526	1.48	0.28
**NBTrp**	n.c.	n.c.	n.c.	n.c.	n.c.	n.c.	n.c.
**NBTrp-vdWonly**	n.c.	n.c.	n.c.	n.c.	n.c.	n.c.	n.c.
**Quinazoline**	0.030±0.011	−0.004±0.003	0.020±0.009	−10.145±0.492	0.108	1.71	1.49
**Quinazoline-vdWonly**	0.017±0.009	/	/	−8.650±0.378	0.039	0.64	0.05

[a] arithmetic mean of the difference between ΔG_{pred} for the full model and all the individual ΔG_{pred} values of the loo models.

Table A2 Parameters for other LIECE models derived from each data set, without an intercept.

	no INT					
	α	β	γ	R^2	RMSE	$\langle\Delta\Delta G\rangle^a$
**DOCK-inactRisp**						
**2Cdrugs**	−0.052±0.037	−0.008±0.013	−0.118±0.026	0.107	1.41	3.64
**2Cdrugs-vdWonly**	0.310±0.003	/	/	0.218	1.69	0.02
**NBTrp**	0.119±0.007	0.020±0.004	−0.037±0.005	0.006	1.16	0.00
**NBTrp-vdWonly**	0.245±0.001	/	/	0.000	1.37	0.03
**Quinazoline**	0.103±0.010	−0.026±0.003	−0.088±0.006	0.455	0.69	0.01
**Quinazoline-vdWonly**	0.259±0.003	/	/	0.013	1.27	0.01
**HYBRID-inactRisp**						
**NBPhe**	0.002±0.018	−0.036±0.023	−0.172±0.026	0.618	1.68	0.28
**NBPhe-vdWonly**	0.353±0.005	/	/	0.075	2.69	0.02
**NBTrp**	−0.035±0.007	−0.004±0.004	−0.119±0.005	0.003	1.11	0.01
**NBTrp-vdWonly**	0.283±0.001	/	/	0.064	1.85	0.01
**Quinazoline**	−0.017±0.007	−0.034±0.003	−0.127±0.003	0.004	1.13	0.02
**Quinazoline-vdWonly**	0.221±0.004	/	/	0.097	4.79	0.05
**DOCK-inactKeta**						
**NBTrp**	0.193±0.009	−0.021±0.002	−0.029±0.006	0.078	1.06	0.00
**NBTrp-vdWonly**	0.236±0.001	/	/	0.002	1.12	0.00
**Quinazoline**	0.108±0.008	0.010±0.002	−0.070±0.005	0.214	0.74	0.02
**Quinazoline-vdWonly**	0.228±0.002	/	/	0.097	1.18	0.01
**HYBRID-inactKeta**						
**NBPhe**	0.177±0.020	−0.084±0.010	−0.139±0.014	0.663	1.26	0.03
**NBPhe-vdWonly**	0.334±0.003	/	/	0.526	1.55	0.01
**NBTrp**	n.c.	n.c.	n.c.	n.c.	n.c.	n.c.
**NBTrp-vdWonly**	n.c.	n.c.	n.c.	n.c.	n.c.	n.c.
**Quinazoline**	−0.001±0.017	−0.037±0.003	−0.124±0.008	0.008	0.62	0.06
**Quinazoline-vdWonly**	0.237±0.002	/	/	0.039	1.78	0.01

[a]arithmetic mean of the difference between ΔG_{pred} for the full model and all the individual ΔG_{pred} values of the loo models.

Table A3 RMSE values of the test set NBPhe, calculated based on the respective LIECE models.

	RMSE (INT)	RMSE (no INT)
**DOCK-inactRisp**		
**NBPhe**	1.14	1.22
**2Cdrugs**	0.92	4.63
**NBTrp**	2.74	2.98
**Quinazoline**	2.39	2.15
**All data sets**	1.79	1.56
**HYBRID-inactRisp**		
**NBPhe**	1.96	0.96
**2Cdrugs**	0.88	0.89
**NBTrp**	3.00	3.48
**Quinazoline**	3.00	4.50
**All data sets**	5.55	5.37
**DOCK-inactKeta**		
**NBPhe**	1.34	1.45
**2Cdrugs**	1.09	2.46
**NBTrp**	4.18	3.54
**Quinazoline**	3.01	2.75
**All data sets**	2.20	1.18
**HYBRID-inactKeta**		
**NBPhe**	1.48	1.22
**2Cdrugs**	1.16	1.12
**NBTrp**	n.c.[a]	n.c.[a]
**Quinazoline**	2.93	3.99
**All data sets**	1.43	1.32

[a]not calculated.

Table A4 ΔG_{pred} values for risperidone, predicted by the respective LIECE models.

	ΔG_{pred} (INT)	ΔG_{pred} (no INT)
**DOCK-inactRisp**		
**2Cdrugs**	−16.85	−14.35
**NBPhe**	−13.51	−16.08
**NBTrp**	−7.92	−13.77
**Quinazoline**	−11.61	−13.75
**All data sets**	−10.91	−15.04
**HYBRID-inactRisp**		
**2Cdrugs**	−19.42	−19.93
**NBPhe**	−37.41	−20.51
**NBTrp**	−6.75	−15.88
**Quinazoline**	−8.79	−13.25
**All data sets**	−16.23	−11.26
**DOCK-inactKeta**		
**2Cdrugs**	−10.69	−19.46
**NBPhe**	−33.17	−18.91
**NBTrp**	−10.69	−9.34
**Quinazoline**	−13.16	−16.81
**All data sets**	−8.57	−18.36
**HYBRID-inactKeta**		
**2Cdrugs**	−21.45	−16.96
**NBPhe**	−23.60	−15.71
**NBTrp**	n.c.[a]	n.c.[a]
**Quinazoline**	−7.76	−13.09
**All data sets**	−10.69	−17.16

[a]not calculated.
